# Rapid optimisation of cellulolytic enzymes ratios in *Saccharomyces cerevisiae* using in vitro SCRaMbLE

**DOI:** 10.1186/s13068-020-01823-8

**Published:** 2020-11-03

**Authors:** Elizabeth L. I. Wightman, Heinrich Kroukamp, Isak S. Pretorius, Ian T. Paulsen, Helena K. M. Nevalainen

**Affiliations:** 1 Centre of Excellence in Synthetic Biology, Department of Molecular Sciences, Macquarie University, Sydney, NSW 2109 Australia; 2 Biomolecular Discovery and Design Research Centre, Macquarie University, Sydney, NSW 2109 Australia; 3 Chancellery, Macquarie University, Sydney, NSW 2109 Australia

**Keywords:** SCRaMbLE, *Saccharomyces cerevisiae*, Consolidated bioprocessing, Cellulases, Enzyme ratios

## Abstract

**Background:**

For the economic production of biofuels and other valuable products from lignocellulosic waste material, a consolidated bioprocessing (CBP) organism is required. With efficient fermentation capability and attractive industrial qualities, *Saccharomyces cerevisiae* is a preferred candidate and has been engineered to produce enzymes that hydrolyze cellulosic biomass. Efficient cellulose hydrolysis requires the synergistic action of several enzymes, with the optimum combined activity ratio dependent on the composition of the substrate.

**Results:**

In vitro SCRaMbLE generated a library of plasmids containing different ratios of a β-glucosidase gene (*CEL3A*) from *Saccharomycopsis fibuligera* and an endoglucanase gene (*CEL5A*) from *Trichoderma reesei*. *S. cerevisiae*, transformed with the plasmid library, displayed a range of individual enzyme activities and synergistic capabilities. Furthermore, we show for the first time that 4,6-O-(3-ketobutylidene)-4-nitrophenyl-β-d-cellopentaoside (BPNPG5) is a suitable substrate to determine synergistic Cel3A and Cel5A action and an accurate predictive model for this synergistic action was devised. Strains with highest BPNPG5 activity had an average *CEL3A* and *CEL5A* gene cassette copy number of 1.3 ± 0.6 and 0.8 ± 0.2, respectively (ratio of 1.6:1).

**Conclusions:**

Here, we describe a synthetic biology approach to rapidly optimise gene copy numbers to achieve efficient synergistic substrate hydrolysis. This study demonstrates how in vitro SCRaMbLE can be applied to rapidly combine gene constructs in various ratios to allow screening of synergistic enzyme activities for efficient substrate hydrolysis.

## Background

Biofuels, made from renewable lignocellulosic biomass, are an attractive alternative to fossil fuels, however, more economic production methods are required. One strategy is to harness a consolidated bioprocessing (CBP) organism which would produce and secrete enzymes that hydrolyze cellulosic material, efficiently carry out fermentation, and be suitable for use at an industrial scale [1, 2]. The yeast *Saccharomyces cerevisiae* is a well-suited CBP candidate that fulfils the latter two requirements, however it does not produce the enzymes required for biomass hydrolysis. The conversion of cellulose into fermentable sugars requires the synergistic action of three main classes of enzymes including β-glucosidase (BGL), endoglucanase (EG) and cellobiohydrolase (CBH) which must be introduced to *S. cerevisiae* [3].

There have been continued efforts towards constructing CBP-ready *S. cerevisiae* strains by sourcing suitable genes from cellulolytic organisms such as *Trichoderma reesei*, *Saccharomycopsis fibuligera, Clostridium thermocellum* and *Aspergillus aculeatus* [4–6]. The construction of these strains has mainly followed the strategies of either producing secreted enzymes or cell-wall tethered enzymes (both individually bound and assembled in synthetic mini cellulosomes) (reviewed by [7]). As a result, *S. cerevisiae* strains capable of simultaneous saccharification and fermentation (SSF) on a range of simple cellulosic substrates are available [8–10], but the ratios of the enzymes required for optimal hydrolysis changes depending on the available substrate and mostly remains undefined [11]. Therefore, many efforts have now turned to the optimisation of enzyme ratios by harnessing different promoters [12], combining specific ratios of yeast strains displaying different enzymes [13] and manipulating gene copy numbers one by one [14].

There have been many rational engineering approaches to enhance cellulase production and secretion in *S. cerevisiae* (reviewed in [15]), however, since the best gene ratio to produce efficient hydrolysis for each substrate is not always known, techniques that generate large libraries of DNA and/or strains containing different gene ratios provide a promising approach. In previous work, Yamada et al. [16] produced a library of cellulolytic yeast strains using cocktail δ-integration. *A. aculeatus CEL3A* (encoding BGLI) and *T. reesei CEL5B* and *CEL6A* (encoding EGII and CBHII, respectively) were randomly integrated into yeast chromosomes in one step, and strains with high enzyme activity on PASC (phosphoric acid swollen cellulose) were obtained. A strain was isolated that contained 1, 13 and 6 copies of *CEL3A*, *CEL5B* and *CEL6A*, respectively, which achieved a PASC degradation activity of 64.9 mU per gramme of wet cell weight. In highlighting the importance of optimal gene ratio rather than overexpression, this strain exhibited higher enzyme activity than the rationally engineered strain, despite having fewer enzyme-encoding gene copies. However, this approach may not give an accurate representation of the most ideal ratio of enzymes, as genes could be integrated in chromosome locations where expression is minimal (e.g. near transcriptionally repressed regions of the telomeres) [17].

A synthetic biology tool which could be used to develop CBP *S. cerevisiae* strains is SCRaMbLE (synthetic chromosome rearrangement and modification by loxPsym-mediated evolution) which facilitates accelerated genome evolution by initiating large-scale DNA recombination in vivo [18]. While “in vivo*”* SCRaMbLE is limited to SCRaMbLE-enabled synthetic chromosomes within the ‘Yeast 2.0’ initiative [18], the in vitro SCRaMbLE of DNA in a test tube generates diverse plasmid libraries that are not only compatible with Yeast 2.0, but can be transformed into any host including *E. coli* and non-synthetic yeasts [19]. In addition, host strains do not require DNA for expression of Cre recombinase, or loxPsym sites required for in vivo SCRaMbLE. Considering this and the fact that in vitro SCRaMbLEd DNA is intended to be maintained as plasmids in the cell, the host genome remains unaltered, retaining any valuable phenotypes of the strain, a feature of importance especially concerning industrial yeasts*. *In vitro SCRaMbLE has been used for *S. cerevisiae* in the optimisation of pathways producing β-carotene and violacein, revealing valuable genotype-to-phenotype relationships [19, 20].

Here, in vitro SCRaMbLE was applied to generate a plasmid library containing different ratios of the β-glucosidase gene *CEL3A* from *S. fibuligera* and the endoglucanase gene *CEL5A* from *T. reesei*. The DNA library was directly transformed into *S. cerevisiae* and synergistic enzyme activity of the recombinant strains was determined using BPNPG5 (Megazyme® K-CellG5-4V) as a substrate for the first time, enabling rapid screening of the transformants. By harnessing the strategy of in vitro SCRaMbLE, coupled with the screening method presented here, the copy number and consequent activity ratio of CEL3A and CEL5A to confer the most efficient activity on BPNPG5 was determined.

## Results

### Plasmid library generation by in vitro SCRaMbLE

**Fig. 1:**
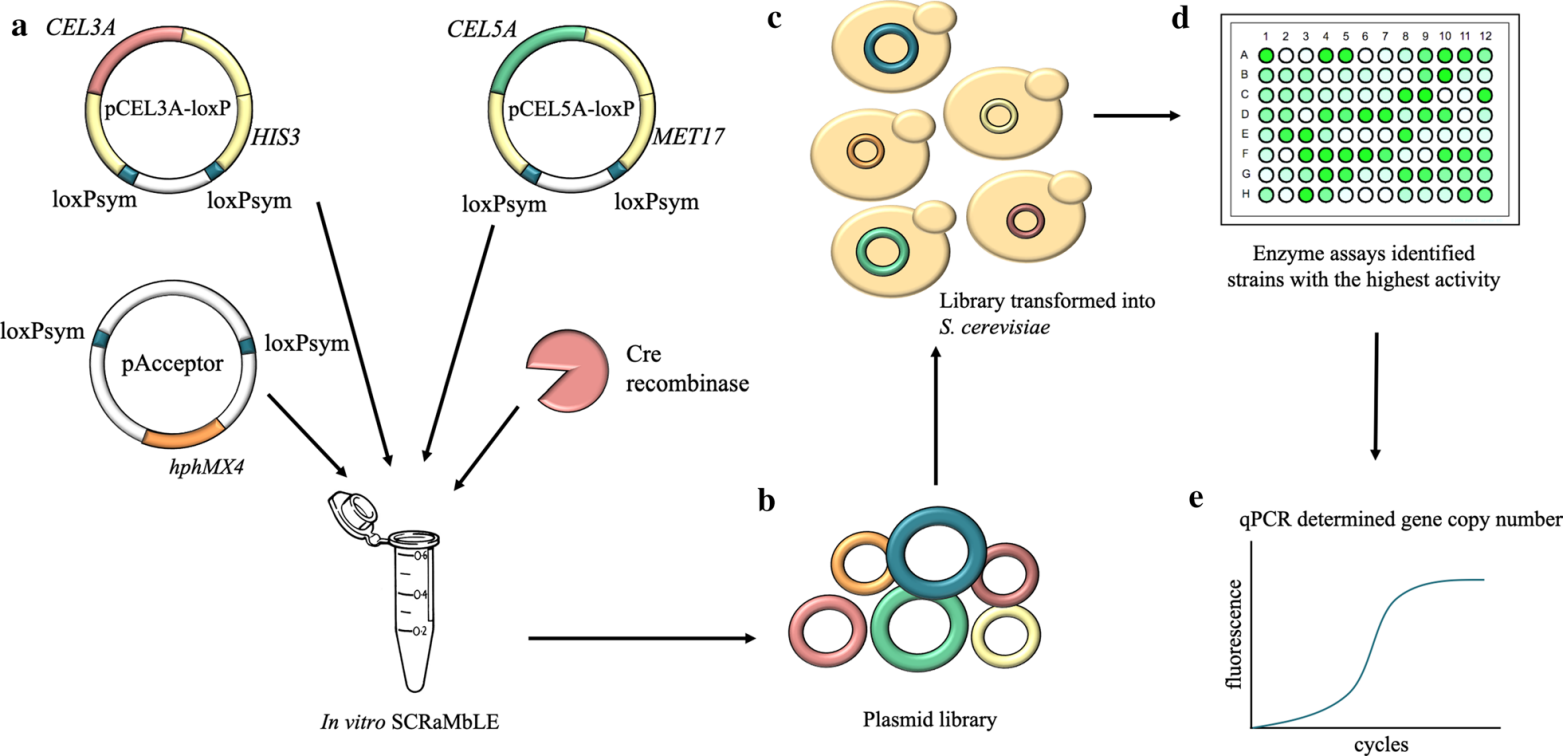
Schematic of the in vitro SCRaMbLE strategy to optimise the gene copy number and ratio of *CEL3A* and *CEL5A* for efficient synergistic enzyme activity. A DNA library was generated by SCRaMbLEing donor DNA (loxPsym-flanked sequences in pCEL3A-loxP and pCEL5A-loxP) in acceptor plasmids (pAcceptor) **(a**). The resulting library **(b**) contained plasmids with various copy numbers of *CEL3A* and *CEL5A* for direct transformation into *S. cerevisiae* (**c**) which conferred a range of synergistic cellulase activity, determined utilising the BPNPG5 substrate (**d**). The copy number of *CEL3A* and *CEL5A* genes which enabled the highest enzyme activity was determined by quantitative PCR (**e**)

The in vitro SCRaMbLE strategy applied for engineering cellulolytic *S. cerevisiae* strains is summarised in Fig. 1. A library of plasmids was constructed by in vitro SCRaMbLEing loxPsym-flanked *CEL3A-HIS3* and *CEL5A-MET17* cassettes into acceptor plasmids (pAcceptor) containing loxPsym sequences and *hphMX4* conferring hygromycin resistance. The generated plasmid library was directly transformed into *S. cerevisiae* BY4741 without plasmid enumeration in *Escherichia coli*. All of the 160 randomly picked putative yeast transformants, selected on SC^−his −met^ supplemented with 200 μg mL^−1^ Hygromycin B agar plates, were confirmed to contain the pAcceptor vector and at least one copy of the *CEL3A* and *CEL5A* expression cassettes using PCR.

### Cellulolytic activity of the *S. cerevisiae* transformants

**Fig. 2:**
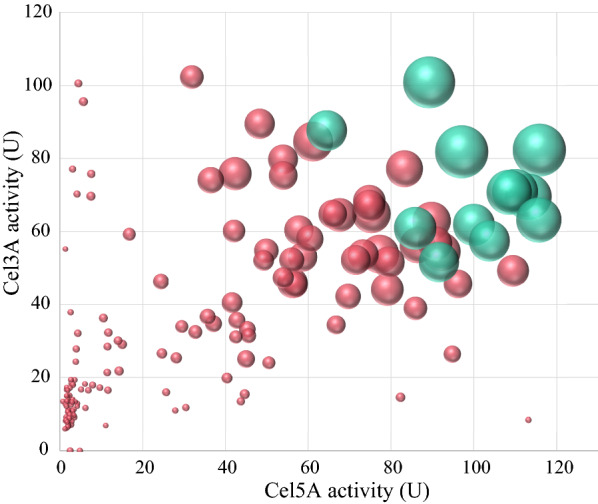
The relationship between Cel5A and Cel3A activity compared to BPNPG5 activity in *S. cerevisiae* strains containing in vitro SCRaMBLEd plasmids. After 48 h of cultivation, the culture supernatant of 160 strains containing in vitro SCRaMbLEd plasmids was used for enzyme assays; Cel3A activity was measured on *p*NPG (*y*-axis), Cel5A activity was measured using a DNS assay on CMC (*x*-axis) and the synergistic activity of both enzymes was measured on BPNPG5 substrate (diameter of spheres). To highlight 12 strains with the highest synergistic enzyme activity, spheres were coloured green

**Fig. 3:**
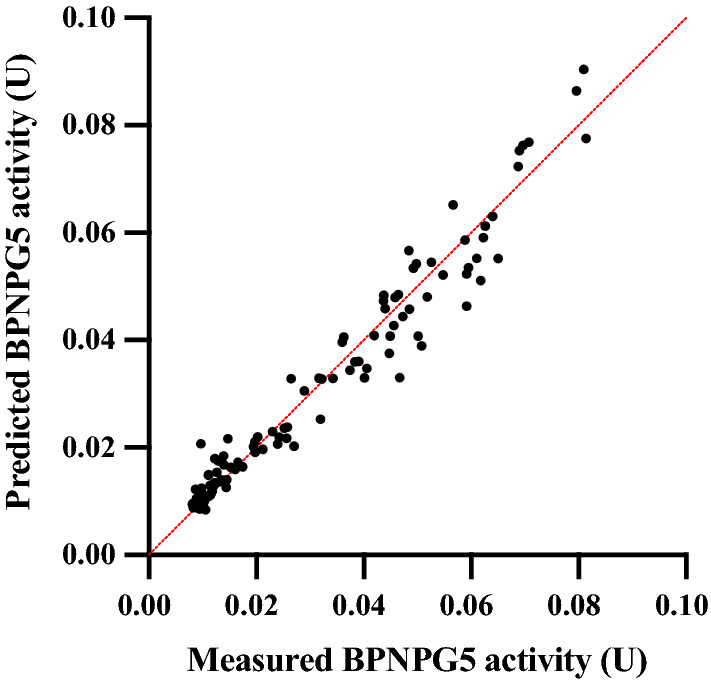
The predicted and actual (measured) BPNPG5 activity of *S. cerevisiae* strains producing Cel3A and Cel5A. All 160 randomly selected strains containing in vitro SCRaMbLEd plasmids were cultivated for 48 h and the culture supernatants were used for enzyme assays; Cel3A activity was measured on *p*NPG, Cel5A activity was measured with DNS assay on CMC and the synergistic activity was measured on BPNPG5. Predicted synergistic activity was calculated using an equation generated by multiple linear regression analysis. Compared to the actual (measured) BPNPG5 activities, this model was highly effective in predicting BPNPG5 activity based on Cel3A and Cel5A (*R*^2^ = 0.97)

The individual and synergistic supernatant enzyme activity of Cel3A and Cel5A produced by the 160 randomly selected *S. cerevisiae* colonies were determined to obtain a comprehensive understanding of the diversity generated by the SCRaMbLEd plasmid library. A wide range of Cel3A and Cel5A enzymatic activities were observed for the evaluated strains, ranging between 0–115.86 ± 8.74 U/mL and 0–102.32 ± 5.20 U/mL, respectively, with a subset of individual strains having both high Cel3A and Cel5A activity (Fig. 2). In general, strains exhibiting both high Cel3A and Cel5A activity also had high BPNPG5 activity (Fig. 2 sphere diameter). Multiple regression analysis confirmed a strong correlation (*R*^2^ = 0.97) between BPNPG5 activity and the combined activities of the Cel3A and Cel5A (Fig. 3). Twelve strains exhibiting the highest BPNPG5 activity were selected for further analysis. Enzyme activity data of these strains are shown in Table 1 and are indicated as green spheres in Fig. 2.

**Table 1:** Enzyme activity of 12 *S. cerevisiae* strains that had the highest synergistic activity on BPNPG5

Strain no.	Cel3A activity (U/mL)	Cel5A activity (U/mL)	BPNPG5 activity (mU/mL)
1	97.1 ± 8.7	81.8 ± 3.45	81.3 ± 3.69
2	115.9 ± 8.7	82.2 ± 5.41	80.9 ± 1.38
3	89.2 ± 1.9	100.9 ± 0.16	79.6 ± 1.90
4	113.2 ± 6.8	69.4 ± 2.13	70.7 ± 5.83
5	110.0 ± 1.8	70.8 ± 3.30	69.7 ± 3.01
6	108.5 ± 3.0	70.7 ± 0.905	68.9 ± 0.14
7	115.7 ± 11.4	63.1 ± 2.21	68.8 ± 0.41
8	85.9 ± 2.2	60.8 ± 2.35	65.0 ± 2.24
9	100.0 ± 4.0	61.8 ± 5.36	64.0 ± 0.24
10	103.8 ± 4.2	57.3 ± 2.64	62.6 ± 0.78
11	64.4 ± 7.3	87.6 ± 4.68	62.2 ± 0.61
12	91.6 ± 2.7	51.6 ± 2.45	61.7 ± 2.44

### BPNPG5 as a substrate to measure synergistic activity of Cel3A and Cel5A

**Fig. 4:**
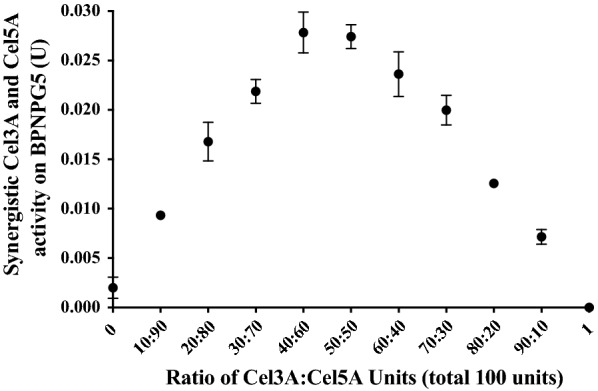
Supernatant enzyme activity on BPNPG5 using mixtures of Cel3A and Cel5A at different ratios. Enzyme mixtures were prepared using the culture supernatants of two *S. cerevisiae* strains producing Cel3A and Cel5A. Activity (*U*) of Cel3A and Cel5A was measured using *p*-NPG and DNS assays, respectively, and mixtures of each were prepared using various ratios of Cel3A and Cel5A activity units from 0 to 1 in increments of 0.1 while the total number of activity units was 100 in all samples

The synergistic action of incremental Cel3A and Cel5A activity changes on BPNPG5 hydrolysis, and the optimum activity ratio were determined (Fig. 4). No detectible *p*NP release were observed from Cel3A activity alone, with some activity detected in the presence of Cel5A only (0.002 ± 0.0009 U). At the respective lowest evaluated Cel3A and Cel5A ratios, namely 10:90 and 90:10, the presence of both enzymes resulted in significantly higher *p*NP release than the sum of the individual actions of the enzymes on BPNPG5 (*p* < 0.001). Maximum BPNPG5 hydrolysis was achieved with Cel3A:Cel5A ratios of 40:60 and 50:50 (enzyme activities using these two ratios were not significantly different, *p* > 0.8) and the following equation was generated to model the action of Cel3A and Cel5A on the BPNPG5 substrate with an *R-*squared value of 0.9658 with 156 degrees of freedom:

$$Y~{\text{ = }}~{\text{8}}{\text{.305e}} - {\text{3}}\;{\text{ + }}\;~{\text{4}}{\text{.1e}} - {\text{5*}}A~\;{\text{ + }}\;{\text{6e}} - {\text{6*}}B\;{\text{ + }}\;{\text{7}}{\text{.594e}} - {\text{6*}}A{\text{*}}B$$

where *Y* = synergistic enzyme activity of Cel3A and Cel5A (*U*), *A* = Cel3A activity (*U*), *B* = Cel5A activity (*U*), significance statistics are listed in Table 2. The ability to produce *p*-nitrophenol from BPNPG5 is highly dependent on synergy between Cel3A and Cel5A.

**Table 2:** *p*-values of variable used in the multiple regression analysis

Variable	*p*-value
Intercept	< 0.0001
*A*	0.0526
*B*	0.0016
A*B	< 0.0001

### Determining *CEL3A* and *CEL5A* cassette ratios conferring high BPNPG5 activity

**Fig. 5:**
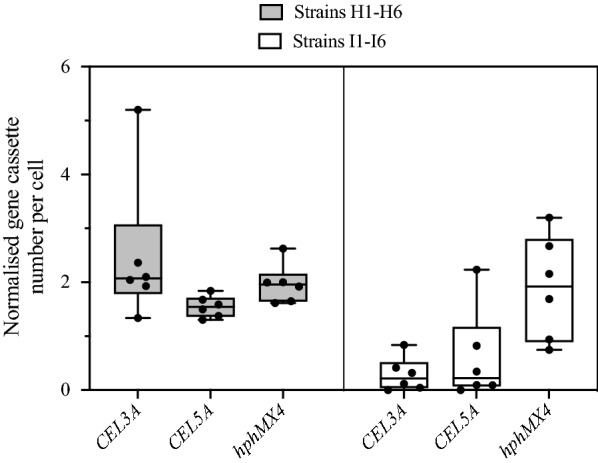
Average copy number of *CEL3A*, *CEL5A* and *hphMX4* in populations of *S. cerevisiae* containing in vitro SCRaMbLEd plasmids. Strains H1–6 (high BPNPG5 activity group) exhibited the highest enzyme activity and strains I1–6 (intermediate BPNPG5 activity group) exhibited approximately the median enzyme activities. The box represents the 25th to 75th percentiles and the line across the box represents the median. The copy number per cell of *CEL3A *and *CEL5A* was significantly greater in strains H1–H6 (*p* < 0.02, *p* < 0.05, respectively) while there was no difference in the copy number of the *hphMX4* marker (*p* > 0.05), suggesting that plasmid copy number was consistent between groups. All gene copy numbers are reported per cell by comparison to a reference gene (*TAF10*) which occurs as a single copy in the haploid genome

The ratio and copy numbers of *CEL3A* and *CEL5A* on in vitro SCRaMbLEd plasmids in *S. cerevisiae* were determined by qPCR (Fig. 5). Two sets of strains were analysed; the ‘high activity’ group contained 6 strains that showed the highest BPNPG5 activity and the ‘intermediate activity’ group which contained 6 strains exhibiting enzyme activities which had approximately the median BPNPG5 activity. The average ratio of *CEL3A* to *CEL5A* genes in strains with the highest BPNPG5 activity was ~ 1.6:1. This ratio was not significantly different to the ratio observed in strains from the intermediate activity group (*p* > 0.2). However, there was a significant difference in gene copy number between the high and intermediate activity groups; the copy numbers of *CEL3A* and *CEL5A* in the high group were 1.3 ± 0.6 and 0.8 ± 0.2, respectively, and in the intermediate group were 0.12 ± 0.10 and 0.26 ± 0.35, respectively. There was a significant difference in copy number for both *CEL3A* high and intermediate groups (*CEL3A*: *p* < 0.01) and for *CEL5A* (*p* < 0.03). There was no significant difference between the plasmid copy number per cell (represented by the *hphMX* cassette abundance, Fig. 5) between the high activity and intermediate activity groups (*p* > 0.2).

## Discussion

In the pursuit of economic bioproduct production from renewable lignocellulosic waste sources, consolidated bioprocessing is considered essential [2]. Significant progress has been made over the last three decades by engineering yeast and other organisms with synthetic cellulolytic and hemi-cellulolytic capabilities (reviewed in [21]). With the major hydrolytic activities achieved in yeast independently, the focus in recent years has shifted to combinatorial cellulolytic enzyme expression and improving substrate utilisation efficiencies [10, 16, 22], with much interest in harmonising the synergistic action of the different enzymatic activities [11]. In this study, we aimed to develop a rapid cellulase expression optimisation system in yeast, allowing selection based on the highest synergistic enzymatic activities.

The generation of expression vectors with randomised copies of *CEL3A* and *CEL5A* was achieved through a simple in vitro assembly strategy where loxP-flanked expression cassettes were combined with a suitable acceptor vector. Similar to random gene integration approaches, our in vitro SCRaMbLE strategy allows the evaluation of gene combinations where enzyme interdependencies or synergies exist, with the additional benefits of an easily scalable DNA assembly step and the capability to generate much greater combinatorial variations. Furthermore, the transformed in vitro SCRaMbLE library is not impacted by chromatin fluidity of the integration site which may impact gene transcription level variations. In our focussed screen within strains with high synergistic enzymatic activity, insertion of up to four expression cassettes was achieved (strain H4, data not shown). This is similar to a previous study which reported up to five insertions for *β*-carotene synthesis pathway assembly, using a similar methodology [19]. In the previous study and this work, only a single round of in vitro SCRaMbLEing was performed; the potential of combinatorial assembly of a wider range of gene cassettes or subsequent rounds of in vitro SCRaMbLE holds the promise of achieving even higher insertion rates.

To facilitate the efficient screening of the combinatorial vector library, a rapid method was developed utilising the BPNPG5 substrate to evaluate the synergistic enzyme activity of Cel3A and Cel5A. While BPNPG5 has previously been used to measure the activity of endoglucanase activity [23], this is to our knowledge the first time BPNPG5 has been used to assess synergistic Cel3A and Cel5A activity.

Our modelling results showed that maximum activity occurred at a Cel3A to Cel5A activity ratio of between 40:60 and 50:50 (Fig. 4), however not all library strains displaying this ideal activity ratio had high BPNPG5 hydrolysis. Up to a limit, the more total enzymatic activity that was present, in the ideal ratio, the higher the BPNPG5 hydrolysis was (Fig. 2). This observation is in line with the basic principles of enzyme kinetics. The impact of enzyme activity levels on BPNPG5 hydrolysis was clearly shown by the comparative BPNPG5 activities of the selected “high activity” and “intermediate activity” groups. Both groups had a similar Cel3A and Cel5A activity ratio (*p* > 0.1), however the high activity group had significantly higher Cel3A and Cel5A activities (*p* < 0.01, *p* < 0.02, respectively) and thus represented library strains with both the ideal gene ratio and optimised enzyme levels.

Reflecting the higher enzymatic activities of the “high activity” group, significantly higher numbers of cellulase expression cassettes were detected per cell, than the corresponding “intermediate activity” group. Strains with the highest BPNPG5 activity had on average 1.3 ± 0.6 *CEL3A* and 0.8 ± 0.2 *CEL5A* cassettes per cell. Between the two selected groups, vectors with one to four cellulase cassette inserts were observed (corresponding to up to 21,158 bp), however this did not have a detectable negative impact on plasmid copy numbers, with all strains having approximately two plasmids per cell (Fig. 5). Although no significant variation in plasmid copy numbers were observed between cells containing different vector sizes in our study, it is reasonable to expect that the incorporation of more DNA, and the metabolic burden of subsequent increased protein production, could impact the overall plasmid copy number per cell [24]. This burden might have been minimised by the unexpected, but previously observed [25], low episomal plasmid copy numbers per cell.

Many factors are at play that influence the optimal ratio of enzyme-encoding genes on substrate hydrolysis. The strategy presented here harnessing in vitro SCRaMbLE efficiently produces diverse libraries of randomised gene-copies in a standardised fashion that can be rapidly screened to uncover optimal ratios for substrates of different compositions. Up to date, rational engineering approaches to optimise enzyme ratios of cellulolytic strains have been limited due to the complexity brought about by (1) gene expression levels, (2) the specific activity and mode of action of enzymes from different origins, (3) the consortium of enzymes used and their relevant synergistic enzyme kinetics, and (4) whether genes will be integrated or maintained on expression plasmids. In a semi-rational screening approach, different optimal gene ratios were found using the cocktail δ-integration method. A *S. cerevisiae* strain with high activity on PASC contained 1, 13 and 6 copies of *Aspergillus aculeatus CEL3A*, *T. reesei CEL5A* and *T. reesei CEL6A* (cellobiohydrolase II-encoding gene), respectively [16]. The differences in gene copy number (compared to the results described here) are not surprising, as a *CEL3A* from a different origin was used (from *A. aculeatus*), the ratio was optimised for activity on a different substrate (PASC) and the action of an addition enzyme was used (Cel6A). Additionally, δ-integration relies on targeting gene cassettes to random retro-transposon sequences throughout the yeast genome, allowing the insertion into genomic regions where expression might be limited or actively silenced (such as near telomeres) and thus might not indicate the ‘ideal’ cassette copy number required for re-engineering purposes. While two cellulase-encoding gene cassettes were used here in this proof-of-concept study, any enzyme-encoding genes could be added to the in vitro SCRaMbLE strategy where specific synergy optimisation between activities is required. In a CBP context, other genes such as those encoding cellobiohydrolases (CBH) or other supplementary activities could be added to enable the hydrolysis of more recalcitrant cellulose substrates such as Avicel. In the interest of uncovering precise ratios for efficient activity, the use of weaker promoters could allow smaller incremental changes and an even more gradual evaluation of expressed enzyme synergistic action.

## Conclusions

The economic production of fuels or other chemicals from biomass requires the efficient conversion of all complex polysaccharides into fermentable sugars. Optimising the ratios of the different hydrolytic activities of enzymes produced by recombinant yeast could achieve efficient release of sugars. In this study, we utilised BPNPG5 as a novel substrate to explore the synergistic action of an endo- and β-glucosidase produced in yeast. Using this substrate, we then proceeded to showcase in vitro SCRaMbLE as a novel synthetic biology tool to rapidly generate large combinatorial plasmid libraries for the co-expression of recombinant cellulases and ultimately demonstrated the capability to use this highly tuneable method to optimise potentially any enzyme system requiring synergy for direct transformation into suitable cell factories for screening.

## Materials and methods

### Strains and media

The yeast strain used in this study was *Saccharomyces cerevisiae* BY4741 (MATa, *his3Δ1*, *leu2Δ0*, *met17Δ0*, *ura3Δ0*) [26]. Cultivation was carried out in liquid or solid (20 g L^−1^ w/v agar) YPD (10 g L^−1^ w/v yeast extract, 20 g L^−1^ w/v peptone, 20 g L^−1^ w/v glucose). Selection medium was 1.7 g L^−1^ yeast nitrogen base without amino acids and ammonium sulphate (Millipore), 1 g L^−1^ L-glutamic acid monosodium salt monohydrate (Sigma-Aldrich), 20 g L^−1^ w/v glucose, 120 mg L^−1^ L-leucine (Sigma-Aldrich), 20 mg L^−1^ uracil (Sigma-Aldrich), 200 µg ml^−1^ Hygromycin B (Invivogen) and 20 g L^−1^ w/v agar. All yeast cultivation was done at 30 °C.

### Cellulase control strain construction

Standard protocols were used for expression vector preparation [27]. Restriction endonucleases, T4 DNA ligase and Phusion DNA polymerase were purchased from Thermo Scientific and used as directed by the manufacturer. The Zymoclean Gel DNA Recovery kit (Zymo Research) was used according to the manufacturer’s instructions to elute digested DNA from agarose gels. For the construction of the episomal cellulase expression plasmids, the Hygromycin B resistance (*hphMX4*) cassette was obtained from the pBHD1_SOD1 plasmid [28] by digesting with *Bam*HI and *Spe*I and subsequent ligation into the pMU1531 [29], creating pHK112. The *Saccharomycopsis fibuligera CEL3A* and *Trichoderma reesei CEL5A* ORFs were amplified from the ySFI vector [30] and pLEGII vector [31], respectively, using primers containing restriction sites for PacI and AscI. These PCR fragments were separated on an agarose gel, purified, digested with PacI and AscI, and cloned into the corresponding sites of the pHK112 vector, yielding pHK112_S.f.CEL3A and pHK112_T.r.CEL5A. The integrity of the constructs were verified with Sanger sequencing (Central Analytical Facility, Stellenbosch, South Africa). The pHK112_S.f.CEL3A and pHK112_T.r.CEL5A vectors were transformed into BY4742 strains [26] using the LiOAc/SS carrier DNA/PEG method as described by Gietz and Schiestl [32] and recovered for 4–6 h in liquid YPD medium at 30 °C and subsequently plated out on YPD agar plates containing 300 µg/mL Hygromycin B. The presence of the expression plasmid in putative transformants was confirmed with polymerase chain reaction (PCR) analysis using the following primers: 5′-GGATCCACTAGTCTTCTAGGCGGGTTATC-3′ and 5′-GACTGGCGCGCCTTACAAACATTGAGAGTAGTATGGG-3′. Confirmed transformants harbouring the pHK112_S.f.CEL3A and pHK112_T.r.CEL5A vectors were referred to as BY4242[Cel3A] and BY4742[Cel5A], respectively.

### SCRaMbLE acceptor plasmid construction

Detailed diagrams of all plasmid maps are provided in the supplementary material. The acceptor plasmid (pAcceptor) was constructed by replacing the cellulase expression cassette with two loxPsym sites in the backbone pHK112 vector. The loxPsym pair was PCR amplified from *S. cerevisiae* synthetic chromosome XIV (unpublished, from our laboratory). The 862 bp between the loxPsym sites contains no known coding sequences. The PCR was performed with 200 µM dNTPs, 0.5 µM of “loxP pair SynXIV F” primer and 0.5 µM of “loxP pair SynXIV R” primer (Table 3), 50 ng of template DNA (gDNA from a yeast strain containing synthetic chromosome XIV), 1 × Phusion^®^ HF buffer (NEB) and 1 unit/50 µl Phusion^®^ High-Fidelity DNA Polymerase (NEB) in a final volume of 50 µl, with the following program: 1 cycle of 98 °C for 30 s, 30 cycles of 98 °C for 30 s, 55 °C for 30 s and 72 °C for 30 s and 1 cycle of 72 °C for 5 min. The primers were designed to produce a PCR product with ends homologous to the plasmid backbone, as such, pAcceptor (Additional file 1: Fig. S1) was constructed using Gibson assembly with the NEBuilder® HiFi DNA Assembly Master Mix (NEB) [33] according to the manufacturer’s instructions.

**Table 3:** Primers used in this study

Primer name	Primer sequence
loxP pair SynXIV F	5′-CCACTAGTCTTCTAGAAAGCATTTTCCCATGAACT-3′
loxP pair SynXIV F	5′-CAAAGAGGTTTAGACGGCCAGGCGTATTCTGATGA-3′
qPCR *CEL5A* F	5′-AGTGGTGTAAGGTTCGCTGG-3′
qPCR *CEL5A* R	5′-GCTGCATTTGACCGATACCG-3′
qPCR *hphMX4* F	5′-CTTCGATGTAGGAGGGCGTG-3′
qPCR *hphMX4* R	5′-TCAGGCTCTCGCTGAATTCC-3′
qPCR *CEL3A* F	5′-GTAGGCTCCAAGACGTCTGG-3′
qPCR *CEL3A* R	5′-CGAATCGAACACCCAATGGC-3′
qPCR *TAF10* F	5′-ATATTCCAGGATCAGGTCTTCCGTAGC-3′
qPCR *TAF10* R	5′-GTAGTCTTCTCATTCTGTTGATGTTGTTGT-3′

### SCRaMbLE donor plasmid construction

The two donor DNA plasmids were chemically synthesised by GenScript, USA. pCEL3A-loxP contained the 2717 bp *S. fibuligera CEL3A* ORF encoding a β-glucosidase I, flanked by the constitutive *TEF1* promoter and the homologous *HXT7* terminator for transcriptional control, followed by *HIS3* as an auxotrophic marker (Additional file 1: Fig. S2). pCEL5A-loxP contained a *S. cerevisiae* codon-optimised 1194 bp T*. reesei CEL5A* encoding endoglucanase with an upstream 57 bp T*. reesei XYN2* signal sequence. *CEL5A* was placed under the transcriptional control of the *PGK1* promoter and the homologous *HXT1* terminator followed by *MET17* as an auxotrophic marker (Additional file 1: Fig. S3). The entire cassettes on both pCEL3A-loxP and pCEL5A-loxP plasmids were flanked by loxPsym sites and reside in the multiple cloning site of a pUC57 cloning plasmid.

### In vitro SCRaMbLE

The bottom-up in vitro SCRaMbLE strategy, described by [19], specifies that loxPsym-flanked donor DNA is SCRaMbLEd into the loxPsym sites in an acceptor plasmid, facilitated by Cre recombinase. In vitro SCRaMbLE was performed as previously described, with adjustments. Briefly, the 50 μl reaction was set up with 1 unit of Cre recombinase (NEB), 400 ng acceptor plasmid, 800 ng pCEL5A-loxP and 800 ng pCEL3A-loxP. Following 1 h incubation at 37 °C, the Cre recombinase enzyme was deactivated at 70 °C for 10 min.

### Transformation and of plasmid library

The library of in vitro SCRaMbLEd plasmids was transformed into BY4741 *S. cerevisiae* [26] as described above. Following transformation, cells were recovered in YPD medium for 4 h, shaking at 200 rpm. Cells were selected on SC^−met −his^ agar supplemented with 200 μg mL^−1^ Hygromycin B to isolate putative transformants which contained pAcceptor and at least one copy of *CEL3A* and *CEL5A*.

### Enzyme assays

Supernatants from 160 *S. cerevisiae* cultures grown for 48 h in YPD supplemented with 200 μg mL^−1^ Hygromycin B were collected for measurement of enzymatic activity. Cel5A activity of culture supernatants was determined by DNS (dinitrosalicylic acid) assay. 10 µl of culture supernatant was incubated with 70 µl of 10 g L^−1^ w/v carboxymethyl cellulose (CMC) in 0.05 M sodium acetate buffer pH 5.0 at 50 °C for 10 min. The addition of 120 µl of DNS reagent [34] was added for determination of reducing sugars. Reactions were boiled and absorbance was measured at 540 nm. For CMC assays, glucose was used to draw a standard curve in the range of 3–50 mM from which the amount of enzymatic Units of each sample was calculated. One Unit of enzyme activity was defined as the amount of enzyme releasing 1 μM of reducing sugar per min. Cel3A activity was determined by the release of *p*-nitrophenol from *p*-nitrophenyl-β-glucoside (*p*NPG). 10 µl of culture supernatant was incubated with 1 µl of 1 mM *p*NPG and 89 µl of 0.05 M Sodium Acetate Buffer pH 5.0, at 50 °C for 10 min. The reaction was stopped with 100 µl of 1 M sodium carbonate and absorbance was measured at 400 nm. One unit of enzyme activity was defined as the amount of enzyme required for producing 1 μM of *p*-nitrophenol from the substrate per min. The synergistic activity of Cel5A and Cel3A was determined by the release of *p*-nitrophenol from BPNPG5 (4,6-O-(3-ketobutylidene)-4-nitrophenyl-β-d-cellopentaoside) obtained from Megazyme. The Cellulase Assay Kit (CellG5 Method) (Megazyme^®^ K-CellG5-4V) was used per the manufacturer’s instructions, except that the addition of β-glucosidase was substituted for 0.05 M acetate buffer. Culture supernatants were incubated with BPNPG5 for 10 min at 37 °C and absorbance was recorded at 405 nm. One unit of enzyme activity was defined as the amount of enzyme required for producing 1 μM of *p*-nitrophenol from the substrate per min. For *p*NPG and BPNPG5 assays a *p*NP standard curve in the range of 1.5–25 mM was used.

### Synergy evaluation and modelling

To evaluate the suitability of BPNPG5 as a substrate to reflect synergistic enzyme activity of Cel3A and Cel5A, supernatants of BY4242[Cel3A] and BY4742[Cel5A] cultures containing either Cel3A or Cel5A, were prepared and the respective enzymatic activities determined using *p*NPG and DNS assays. Supernatants were mixed in different ratios and BPNPG5 hydrolysis evaluated, as described above. Keeping the total enzymatic units constant (at 100 U), the combined action of different ratios (increments of 10%) of Cel3A and Cel5A were determined. To model this synergistic relationship between Cel3A and Cel5A activity a multiple linear regression analysis (least squares) was performed using GraphPad Prism version 8.01 for Windows, GraphPad Software, La Jolla California USA (www.graphpad.com). Based on this model, theoretical BPNPG5 activity were predicted for 160 yeast strains with in vitro SCRaMbLEd plasmids, and compared with measured BPNPG5 activity.

### Extraction of DNA from yeast

Total DNA from yeast transformants was extracted using the dilute sodium hydroxide lysis method. In short, yeast cells 100 μl of an overnight yeast culture, grown in YPD supplemented with 200 µg ml^−1^ Hygromycin B, were pelleted by centrifugation at 4000 rpm for 2 min. The cell pellet was resuspended in 20 mM NaOH. Cell suspensions were incubated at 95 °C for 10 min. After pelleting cell debris by centrifugation at 4000 rpm for 2 min, cell lysates were directly used for quantitative PCR.

### Determination of gene copy number in plasmids

Quantitative real-time PCR (qPCR) was carried out using the Roche LightCycler^®^ 480 instrument. Each reaction contained 5 μl of Agilent Technologies Brilliant II SYBR^®^ Green QPCR Master Mix, 200 nM forward primer, 200 nM reverse primer, 1 µl of DNA template (supernatant from NaOH extractions) and nuclease-free water to obtain a final volume of 10 μl. Four sets of primers were designed to specifically amplify ~ 150 bp of the *CEL3A, CEL5A* and *hphMX4* cassettes on plasmids and the native *TAF10* from the genome (primers are listed in Table 2). Primer amplification efficiencies were determined using serial dilutions of total extracted yeast DNA. The cycling protocol used an initial denaturing step of 95 °C for 1 min, followed by 40 cycles of 95 °C for 30 s, 55 °C for 30 s and 72 °C for 30 s. Fluorescence was recorded following each 72 °C cycling step. Crossing point (Cp) values were calculated using the Absolute Quantification software modules of the LightCycler^®^ 480 Software. The number of plasmids per cell was determined as the relative *hphMX4* copies per sample compared to the relative number of *TAF10* copies of the same sample.

## Supplementary information

**Additional file 1: Fig. S1.** Plasmid map of pAcceptor showing important features. The acceptor plasmid was constructed on a yeast episomal pRS-based plasmid with *hphMX4* conferring hygromycin resistance, and two loxPsym sites. The loxPsym pair was PCR amplified from *S. cerevisiae* synthetic chromosome XIV (unpublished, from our laboratory). The 862 bp between the loxPsym sites contains no known coding sequences.** Fig. S2.** Plasmid map of pCEL3A-loxP showing important features. The 2717 bp *S. fibuligera* *CEL3A* encoding β-glucosidase I was flanked by the homologous, constitutive *TEF1* promoter and the homologous *HXT7* terminator followed by *HIS3* (with native promoter and terminator) as an auxotrophic marker. The entire cassette is flanked by loxPsym sites and resides in the multiple cloning site of a pUC57 cloning plasmid.** Fig. S3.** Plasmid map of pCEL5A-loxP showing important features. pCEL5A-loxP contained a *S. cerevisiae* codon-optimized 1194 bp *T. reesei CEL5A* encoding endoglucanase with an upstream 57 bp *T. reesei xyn2 *secretion signal sequence. *CEL5A* was placed under the expression control of the *PGK1* promoter and the homologous *hxt1* terminator followed by homologous *MET17* (with native promoter and terminator) as an auxotrophic marker. The entire cassette is flanked by loxPsym sites and resides in the multiple cloning site of a pUC57 cloning plasmid.

## Data Availability

Plasmids and strains developed in this study will be made available on request to the corresponding author. Additional data and plasmid maps are provided as supplementary data.

## References

[1] Lynd LR, Weimer PJ, van Zyl WH, Pretorius IS (2002). Microbial cellulose utilization: fundamentals and biotechnology. Microbiol Mol Biol R.

[2] van Zyl WH, Lynd LR, den Haan R, McBride JE, Consolidated bioprocessing for bioethanol production using *Saccharomyces cerevisiae*. In: Biofuels*.* Springer, Berlin. 2007;205–235. 10.1007/10_2007_061 17846725

[3] Srivastava N, Srivastava M, Mishra P, Gupta VK, Molina G, Rodriguez-Couto S, Manikanta A, Ramteke P (2018). Applications of fungal cellulases in biofuel production: advances and limitations. Renew Sust Energ Rev.

[4] Fujita Y, Ito J, Ueda M, Fukuda H, Kondo A (2004). Synergistic saccharification, and direct fermentation to ethanol, of amorphous cellulose by use of an engineered yeast strain codisplaying three types of cellulolytic enzyme. Appl Environ Microbiol.

[5] Jeon E, Hyeon J, Suh DJ, Suh Y, Kim SW, Song KH, Han SO (2009). Production of cellulosic ethanol in *Saccharomyces cerevisiae* heterologous expressing *Clostridium thermocellum* endoglucanase and *Saccharomycopsis fibuligera* β-glucosidase genes. Mol Cells.

[6] Nevalainen KH, The molecular biology of *Trichoderma* and its application to the expression of both homologous and heterologous genes. In: Molecular Industrial Mycology*.* Routledge. 2017;129–148.

[7] den Haan R, van Rensburg E, Rose SH, Görgens JF, van Zyl WH (2015). Progress and challenges in the engineering of non-cellulolytic microorganisms for consolidated bioprocessing. Curr Opin Biotech.

[8] Lee WH, Jin Y (2017). Improved ethanol production by engineered *Saccharomyces cerevisiae* expressing a mutated cellobiose transporter during simultaneous saccharification and fermentation. J Biotechnol.

[9] Lee WH, Nan H, Kim HJ, Jin YS (2013). Simultaneous saccharification and fermentation by engineered *Saccharomyces cerevisiae* without supplementing extracellular β-glucosidase. J Biotechnol.

[10] Claes A, Deparis Q, Foulquié-Moreno MR, Thevelein JM (2020). Simultaneous secretion of seven lignocellulolytic enzymes by an industrial second-generation yeast strain enables efficient ethanol production from multiple polymeric substrates. Metab Eng.

[11] Hu J, Arantes V, Pribowo A, Saddler JN (2013). The synergistic action of accessory enzymes enhances the hydrolytic potential of a “cellulase mixture” but is highly substrate specific. Biotechnol Biofuels.

[12] Song X, Li Y, Wu Y, Cai M, Liu Q, Gao K, Zhang X, Bai Y, Xu H, Qiao M (2018). Metabolic engineering strategies for improvement of ethanol production in cellulolytic *Saccharomyces cerevisiae*. FEMS Yeast Res..

[13] Baek SH, Kim S, Lee K, Lee JK, Hahn JS (2012). Cellulosic ethanol production by combination of cellulase-displaying yeast cells. Enzyme Microb Technol.

[14] Matano Y, Hasunuma T, Kondo A (2012). Display of cellulases on the cell surface of *Saccharomyces cerevisiae* for high yield ethanol production from high-solid lignocellulosic biomass. Bioresour Technol.

[15] Kroukamp H, den Haan R, van Zyl JH, van Zyl WH (2018). Rational strain engineering interventions to enhance cellulase secretion by *Saccharomyces cerevisiae*. Biofuel Bioprod Bior.

[16] Yamada R, Taniguchi N, Tanaka T, Ogino C, Fukuda H, Kondo A (2010). Cocktail δ-integration: a novel method to construct cellulolytic enzyme expression ratio-optimized yeast strains. Microb Cell Fact.

[17] Lustig AJ (1998). Mechanisms of silencing in *Saccharomyces cerevisiae*. Curr Opin Genet Dev.

[18] Dymond J, Boeke J (2012). The *Saccharomyces cerevisiae* SCRaMbLE system and genome minimization. Bioengineered.

[19] Wu Y, Zhu RY, Mitchell LA, Ma L, Liu R, Zhao M, Jia B, Xu H, Li YX, Yang ZM (2018). In vitro DNA SCRaMbLE. Nat Commun.

[20] Liu W, Luo Z, Wang Y, Pham NT, Tuck L, Pérez-Pi I, Liu L, Shen Y, French C, Auer M (2018). Rapid pathway prototyping and engineering using in vitro and in vivo synthetic genome SCRaMbLE-in methods. Nat Commun.

[21] den Haan R, Kroukamp H, Mert M, Bloom M, Görgens JF, Van Zyl WH (2013). Engineering *Saccharomyces cerevisiae* for next generation ethanol production. J Chem Technol Biotechnol.

[22] Oh EJ, Jin YS (2020). Engineering of *Saccharomyces cerevisiae* for efficient fermentation of cellulose. FEMS Yeast Res..

[23] Mangan D, Cornaggia C, McKie V, Kargelis T, McCleary BV (2016). A novel automatable enzyme-coupled colorimetric assay for endo-1, 4-β-glucanase (cellulase). Anal Bioanal Chem.

[24] Karim AS, Curran KA, Alper HS (2013). Characterization of plasmid burden and copy number in *Saccharomyces cerevisiae* for optimization of metabolic engineering applications. FEMS Yeast Res.

[25] Chen Y, Partow S, Scalcinati G, Siewers V, Nielsen J (2012). Enhancing the copy number of episomal plasmids in *Saccharomyces cerevisiae* for improved protein production. FEMS Yeast Res.

[26] Brachmann CB, Davies A, Cost GJ, Caputo E, Li J, Hieter P, Boeke JD (1998). Designer deletion strains derived from *Saccharomyces cerevisiae* S288C: a useful set of strains and plasmids for PCR-mediated gene disruption and other applications. Yeast.

[27] Russell DW, Sambrook J (2001). Molecular Cloning: a laboratory Manual.

[28] Kroukamp H, den Haan R, van Wyk N, van Zyl WH (2013). Overexpression of native *PSE1* and *SOD1* in *Saccharomyces cerevisiae* improved heterologous cellulase secretion. Appl Energy.

[29] Ilmén M, Den Haan R, Brevnova E, McBride J, Wiswall E, Froehlich A, Koivula A, Voutilainen SP, Siika-Aho M, la Grange DC (2011). High level secretion of cellobiohydrolases by *Saccharomyces cerevisiae*. Biotechnol Biofuels.

[30] van Rooyen R, Hahn-Hägerdal B, la Grange DC, Van Zyl WH (2005). Construction of cellobiose-growing and fermenting *Saccharomyces cerevisiae* strains. J Biotechnol.

[31] du Plessis L, Rose SH, van Zyl WH (2010). Exploring improved endoglucanase expression in *Saccharomyces cerevisiae* strains. Appl Microbiol Biotechnol.

[32] Gietz RD, Schiestl RH (2007). High-efficiency yeast transformation using the LiAc/SS carrier DNA/PEG method. Nat Protoc.

[33] Gibson DG, Benders GA, Andrews-Pfannkoch C, Denisova EA, Baden-Tillson H, Zaveri J, Stockwell TB, Brownley A, Thomas DW, Algire MA (2008). Complete chemical synthesis, assembly, and cloning of a *Mycoplasma genitalium* genome. Science.

[34] Miller GL (1959). Use of dinitrosalicylic acid reagent for determination of reducing sugar. Anal Chem.

