# Supplementary material for: Rapid optimisation of cellulolytic enzymes ratios in Saccharomyces cerevisiae using in vitro SCRaMbLE
Source: Biotechnol Biofuels. 2020 Nov 3;13:182. doi: 10.1186/s13068-020-01823-8 (PMC7607656; doi:10.1186/s13068-020-01823-8)
Supplement: Supplementary file 1 — Additional file 1: Fig. S1. Plasmid map of pAcceptor showing important features. The acceptor plasmid was constructed on a yeast episomal pRS-based plasmid with hphMX4 conferring hygromycin resistance, and two loxPsym sites. The loxPsym pair was PCR amplified from S. cerevisiae synthetic chromosome XIV (unpublished, from our laboratory). The 862 bp between the loxPsym sites contains no known coding sequences. Fig. S2. Plasmid map of pCEL3A-loxP showing important features. The 2717 bp S. fibuligera CEL3A encoding β-glucosidase I was flanked by the homologous, constitutive TEF1 promoter and the homologous HXT7 terminator followed by HIS3 (with native promoter and terminator) as an auxotrophic marker. The entire cassette is flanked by loxPsym sites and resides in the multiple cloning site of a pUC57 cloning plasmid. Fig. S3. Plasmid map of pCEL5A-loxP showing important features. pCEL5A-loxP contained a S. cerevisiae codon-optimized 1194 bp T. reesei CEL5A encoding endoglucanase with an upstream 57 bp T. reesei xyn2 secretion signal sequence. CEL5A was placed under the expression control of the PGK1 promoter and the homologous hxt1 terminator followed by homologous MET17 (with native promoter and terminator) as an auxotrophic marker. The entire cassette is flanked by loxPsym sites and resides in the multiple cloning site of a pUC57 cloning plasmid. [file 13068_2020_1823_MOESM1_ESM.docx]

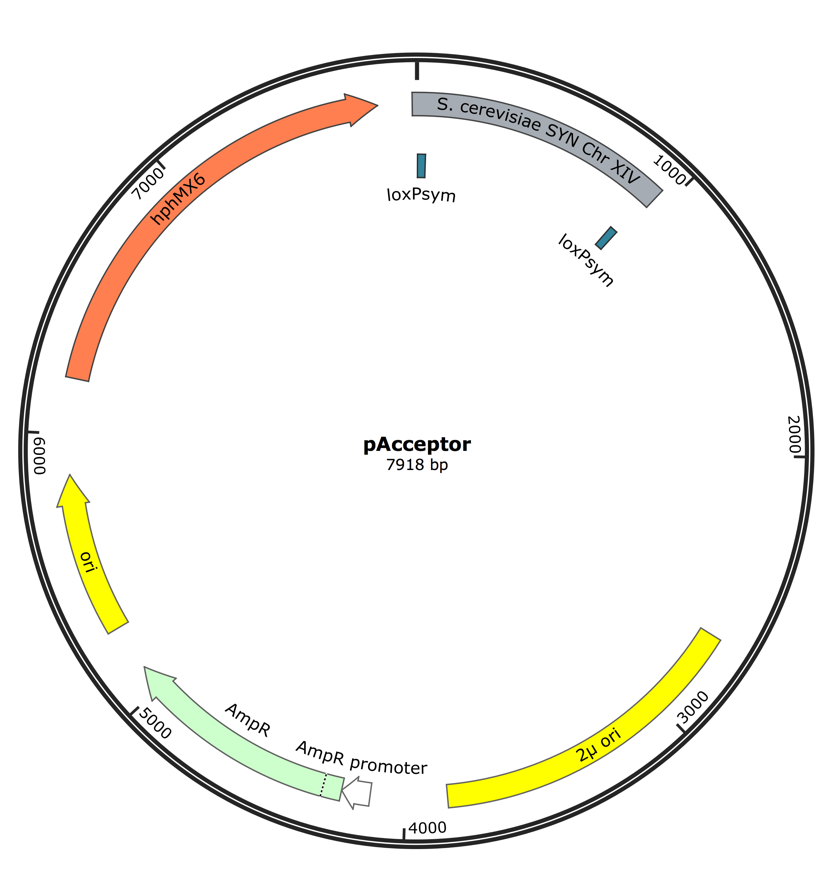
**Additional file 1**

**Fig. S1** Plasmid map of pAcceptor showing important features. The acceptor plasmid was constructed on a yeast episomal pRS-based plasmid with *hphMX4* conferring hygromycin resistance, and two loxPsym sites. The loxPsym pair was PCR amplified from *S. cerevisiae* synthetic chromosome XIV (unpublished, from our laboratory). The 862 bp between the loxPsym sites contains no known coding sequences.


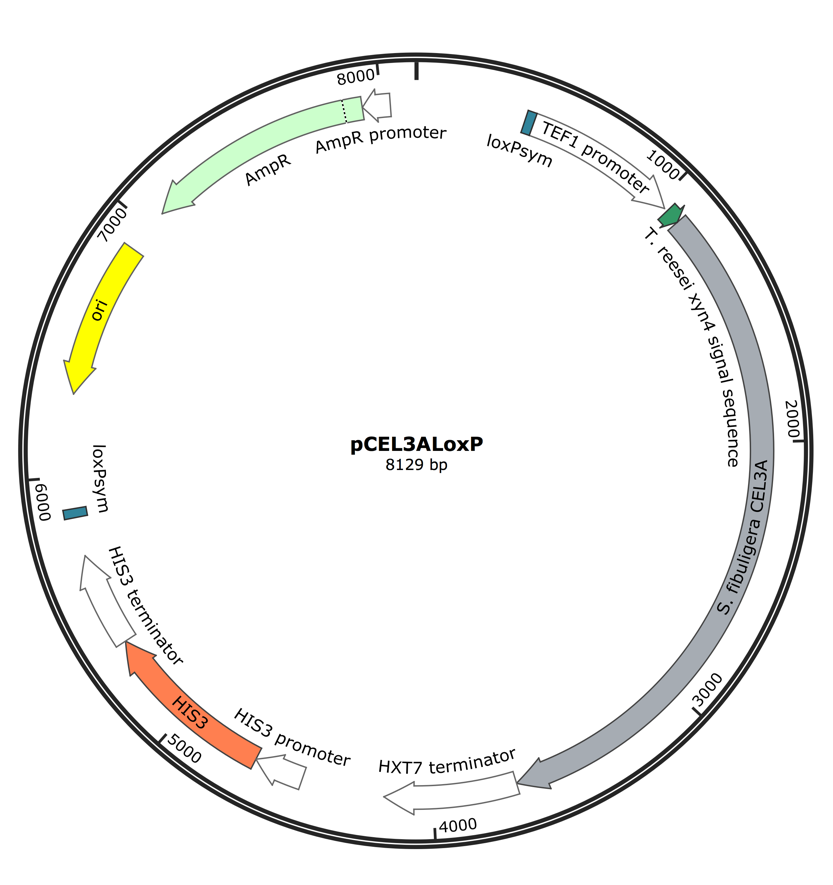


**Fig. S2** Plasmid map of pCEL3A-loxP showing important features. The 2717 bp *S. fibuligera* *CEL3A* encoding β-glucosidase I was flanked by the homologous, constitutive *TEF1* promoter and the homologous *HXT7* terminator followed by *HIS3* (with native promoter and terminator) as an auxotrophic marker. The entire cassette is flanked by loxPsym sites and resides in the multiple cloning site of a pUC57 cloning plasmid.


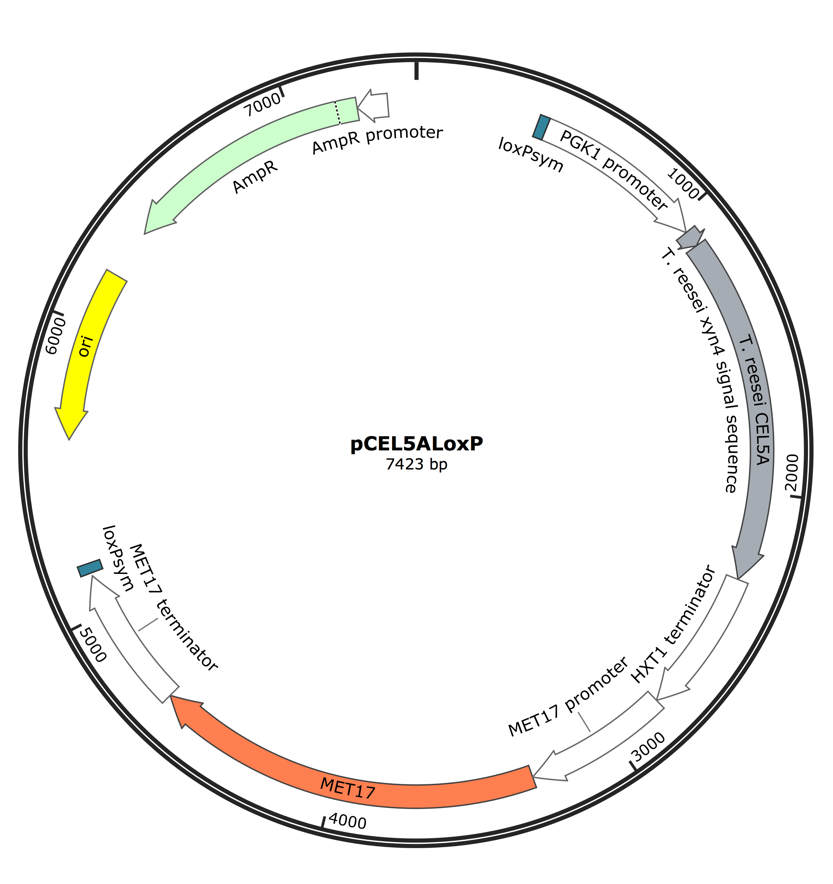


**Fig. S3** Plasmid map of pCEL5A-loxP showing important features. pCEL5A-loxP contained a *S. cerevisiae* codon-optimized 1194 bp *T. reesei* *CEL5A* encoding endoglucanase with an upstream 57 bp *T. reesei* *xyn2* secretion signal sequence. *CEL5A* was placed under the expression control of the *PGK1* promoter and the homologous *hxt1* terminator followed by homologous *MET17* (with native promoter and terminator) as an auxotrophic marker. The entire cassette is flanked by loxPsym sites and resides in the multiple cloning site of a pUC57 cloning plasmid.
